# Impact of COVID-19 outbreak on stroke admission in Thailand: a quasi-experimental, ecological study on national database

**DOI:** 10.1080/20008686.2023.2270261

**Published:** 2023-10-23

**Authors:** Kannikar Kongbunkiat, Donlagon Jumparway, Nisa Vorasoot, Narongrit Kasemsap, Kittisak Sawanyawisuth, Somsak Tiamkao

**Affiliations:** a Division of Neurology, Department of Medicine, Faculty of Medicine, Khon Kaen University, Khon Kaen, Thailand; b North-Eastern Stroke Research Group, Khon Kaen University, Khon Kaen, Thailand; c ASEAN Cancer Epidemiology and Prevention Research Group, Faculty of Public Health, Khon Kaen University, Khon Kaen, Thailand; d Division of Ambulatory Medicine, Department of Medicine, Faculty of Medicine, Khon Kaen University, Khon Kaen, Thailand

**Keywords:** COVID-19, thrombotic, embolic, hemorrhagic, incidence

## Abstract

This study aimed to evaluate the effect of COVID-19 outbreak on stroke admission by using a national database. A quasi-experimental, ecological study using the national database of Thailand was conducted. The study period was between January 2017 and August 2020 before and after COVID-19 outbreak starting from March 2020. Numbers of stroke admission were evaluated before and after the COVID-19 outbreak by an interrupted time series analysis for both pre- and post-COIVD-19 outbreak. There were 381,891 patients admitted throughout Thailand. Of those, 292,382 patients (76.56%) were admitted due to thrombotic stroke followed by hemorrhagic stroke (73,130 patients; 19.15%) and embolic stroke (16,379 patients; 4.29%). During pre-COVID-19 outbreak, all stroke subtypes had an increasing trend with a coefficient of 0.076 (*p* value < 0.001) for thrombotic stroke, 0.003 (*p* value < 0.001) for embolic stroke and 0.012 (*p* value = 0.025) for hemorrhagic stroke. The COVID-19 outbreak had significantly effect on reductions of incidence rates of thrombotic and hemorrhagic stroke with a coefficient of −2.412 (*p* value < 0.001) and −0.803 (*p* value = 0.023). The incidence rates of three stroke subtypes were increasing prior to the COVID-19 outbreak. The COVID-19 outbreak significantly impacts hospital admission rates of both thrombotic and hemorrhagic stroke subtypes.

## Introduction

Stroke is a common neurological disease with 13.7 million new cases and 80.1 million prevalent cases globally in 2016 [1]. It causes 5.5 million deaths and 116.4 million disability-adjusted life-years. Several factors are defined as risk factors for stroke such as hypertension, dyslipidemia or obstructive sleep apnea [2–7]. Coronavirus disease-2019 or COVID-19 is a global pandemic disease. COVID-19 has been reported to increase risks of stroke by causing endothelial injury and hypercoagulability [8,9]. Several reports showed effects of COVID-19 in stroke [10–13]. In the USA, the number of patients underwent brain imaging was decreasing from 1.18 to 0.72 patients/day/hospital after the COVID-19 pandemic [10]. Seven out of 13 stroke care centers in India treated only treated stroke patients who tested negative for COVID-19 [11].

At least two reports from two regional facilities in France and Spain found that COVID-19 pandemic had an impact on stroke [12,13]. In France, stroke admission was somewhat lower by 0.6% without time delays or severity of clinical symptoms between pre- and post-COVID-19 outbreak [12]. Note that stroke alerts were decreasing by 39.6%. In Spain, stroke admission was decreasing by 23% with younger age of stroke admission during COVID-19 pandemic versus pre-pandemic period (69 vs 75 years; *p* = 0.009). There were limited data on stroke admission during COVID-19 outbreak by national database [14]. Therefore, this study aimed to assess the impact of COVID-19 pandemic on stroke care as measured by stroke admission because COVID-19 overwhelmed the healthcare system and non-COVID-19 healthcare was negatively impacted by using a Thai national database.

## Methods

This was a quasi-experimental, ecological study using the Universal Coverage Health Security Insurance Scheme (UCHSIS) database of Thailand [15]. This database is a fundamental, basic health insurance for 75% of Thai population [16]. The UCHSIS database is used for reimbursement purpose for the UCHSIS and not open to public. Data from all levels of hospitals are submitted to the system after the discharge within one month. The attending physicians provided a summary of personal data/national ID number, diagnosis and discharge status. Data of each patient are audit by the authorized persons of UCHSIS for the reimbursement. Note that authorization for reimbursement may be 2 to 3 months after data submission to the system. The inclusion criteria were numbers of adult patients admitted with stroke throughout Thailand regardless results of COVID-19 test. The diagnosis of stroke was made and retrieved from the database using the International Classification of Disease, Tenth Revision (ICD-10) codes. Stroke subtypes were identified and classified as ischemic (I63) and hemorrhagic (I61, I62). Ischemic stroke is classified as thrombotic and embolic. Those with unavailable computed tomography of the brain or undetermined pathology (I64) were excluded. The study period was between January 2017 and August 2020; before and after COVID-19 outbreak. The study protocol was approved by the ethics committee in human researh, Khon Kaen University, Khon Kaen, Thailand (HE631574).

Numbers and incidence rates of patients with stroke were reported and categorized by stroke types and by months admitted. Only new cases of stroke during the study period were identified and counted by using the national ID numbers. Those with recurrent stroke were count as only one patient. The denominator for the incidence calculation was the mid-year numbers of population of Thailand in each year. The COVID-19 outbreak in Thailand was in March 2020 and used as an intervention period. Period before March 2020 was used as pre-intervention period, while period after March 2020 was identified as post-intervention period. The study ended in August 2020 as the new wave of COVID-19 infection in Thailand started in September 2020. Trend of each stroke subtype was evaluated by an interrupted time series analysis for both pre- and post-COVID-19 outbreak [17]. The interrupted time series analysis model was performed to evaluate the effects of COVID-19 outbreak on incidence rate of each stroke subtype. Coefficients with 95% confidence interval and *p* value of pre-intervention period (time variable), intervention and post-intervention (rank) were reported. Ordinary least squares (OLS) were used to assess the magnitude and direction of impacts by using sandwich lmtest and zoo packages as well as important assumption inspections including autocorrelation using car packages in Durbin–Watson testing and ggplot2 package for plot the graph [18–20]. Statistical analyses were performed by STATA software, version 15 (College Station, Texas, USA) and R software, version 4.0.4.

## Results

**Figure 1.**
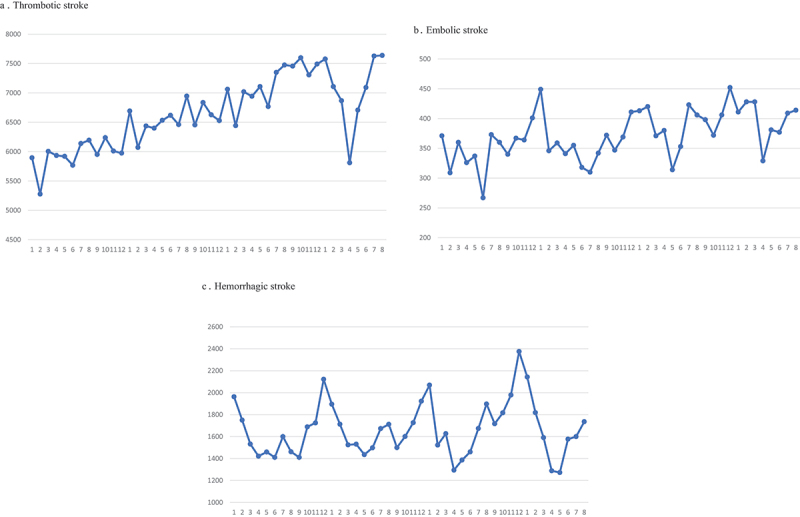
Numbers of admitted stroke patients from January 2017 to August 2020 by types of stroke and months: A: thrombotic; B: embolic; C: hemorrhagic.

During the study period, there were 381,891 patients admitted and reported to the database. Of those, 292,382 patients (76.56%) were admitted due to thrombotic stroke followed by hemorrhagic stroke (73,130 patients; 19.15%) and embolic stroke (16,379 patients; 4.29%) as shown in Table 1. The numbers of thrombotic stroke patients had increasing trend throughout the study period (Figure 1a) and significantly dropped in April 2020, while the numbers of embolic and hemorrhagic stroke had wax and wane pattern (Figure 1b,c).

**Table 1.** Numbers of admitted stroke patients from January 2017 to August 2020 by types of stroke and months.

Year	Month	Thrombotic	Embolic	Hemorrhagic
2017	January	5896	371	1963
	February	5278	309	1750
	March	6005	360	1532
	April	5935	326	1422
	May	5920	337	1460
	June	5769	267	1411
	July	6139	373	1600
	August	6195	360	1462
	September	5952	340	1411
	October	6238	367	1689
	November	6011	364	1725
	December	5975	401	2122
	Total	71313	4175	19547
2018	January	6692	449	1895
	February	6072	346	1713
	March	6437	359	1525
	April	6400	341	1531
	May	6534	355	1436
	June	6619	318	1498
	July	6461	310	1673
	August	6946	342	1712
	September	6455	372	1500
	October	6837	347	1601
	November	6628	369	1727
	December	6529	411	1923
	Total	78610	4319	19734
2019	January	7062	413	2069
	February	6443	420	1523
	March	7020	371	1627
	April	6943	380	1295
	May	7108	314	1387
	June	6768	353	1461
	July	7350	423	1674
	August	7476	406	1897
	September	7457	398	1717
	October	7599	372	1817
	November	7307	406	1979
	December	7492	452	2375
	Total	86025	4708	20821
2020	January	7578	411	2143
	February	7108	428	1819
	March	6868	428	1591
	April	5811	329	1289
	May	6708	381	1273
	June	7093	377	1577
	July	7629	409	1600
	August	7639	414	1736
	Total	56434	3177	13028
Grand total	381,891	292,382	16,379	73,130

**Figure 2.**
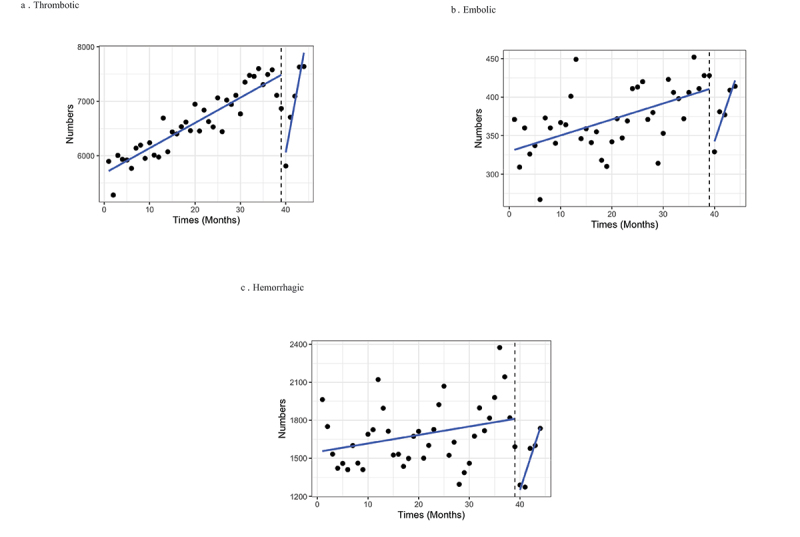
Interrupted time series on numbers of stroke admission by types of stroke before and after the COVID-19 outbreak in March 2020 (dot line).

Figure 2(a to c) shows interrupted time series analysis. After the COVID-19 outbreak in March, 2020 (dot line), all three stroke types had increasing trend. Only thrombotic stroke had significantly increasing trend with a rank coefficient of 0.340 (*p* value < 0.001) as shown in Table 2. During pre-COVID-19 outbreak, all stroke subtypes had an increasing trend with a coefficient of 0.076 (*p* value < 0.001) for thrombotic stroke, 0.003 (*p* value < 0.001) for embolic stroke and 0.012 (*p* value = 0.025) for hemorrhagic stroke. The COVID-19 outbreak had significantly effect on reductions of incidence rates of thrombotic and hemorrhagic stroke with a coefficient of −2.412 (*p* value < 0.001) and −0.803 (*p* value = 0.023) as shown in Table 2.

**Table 2.** Interrupted time series regression model (incident per 100,000 population) shows an analysis of the impact of COVID-19 outbreak on stroke incident rates by stroke types.

			95% confidence interval		
	Variable	Coefficient	Lower	upper	P-value
Thrombotic (*n* = 292,382)					
	(Intercept)	8.532	8.258	8.807	<0.001
	Time	0.076	0.063	0.088	<0.001
	Intervention	−2.412	−3.227	−1.596	<0.001
	Rank	0.340	0.142	0.539	0.001
Embolic (*n* = 16, 379)					
	(Intercept)	0.500	0.464	0.536	<0.001
	Time	0.003	0.001	0.005	<0.001
	Intervention	−0.058	−0.165	0.049	0.283
	Rank	0.004	−0.022	0.030	0.757
Hemorrhagic (*n* = 73130)					
	(Intercept)	2.326	2.094	2.557	<0.001
	Time	0.012	0.002	0.022	0.025
	Intervention	−0.803	−1.492	−0.114	0.023
	Rank	0.072	−0.095	0.240	0.389

## Discussion

This national-based study showed that COVID-19 outbreak had significantly impact on stroke admission for both thrombotic and hemorrhagic stroke subtypes.

As previously reported [13,14], COVID-19 outbreak had negative impact on stroke admission. Even though this larger study showed that all three stroke types had negative coefficients (Table 2), only thrombotic and hemorrhagic stroke subtypes had significant *p* value with a coefficient of −2.412 and −0.803, respectively. These results indicated that COVID-19 outbreak lower incidence rate of thrombotic stroke admission by 2.412 persons/100,000 population, while the incidence of hemorrhagic stroke admission was lower by 0.803 persons/100,000 population. These findings may explain by severity of each stroke type. Thrombotic stroke including lacunar infarction may have less severity resulting in delay in hospital admission. A previous study found that patients with hemorrhagic stroke had more severe stroke score than patients with ischemic stroke significantly (28.3 vs 42.9; p < 0.001). Additionally, hemorrhagic stroke was an independent factor associated with mortality: hazard ratio of 1.564 with 95% CI of 1.441–1.696 [21]. The high mortality rate of hemorrhagic stroke was also explained lower numbers of admission of patients with hemorrhagic stroke as these patients may die at home without hospital admissions.

A previous study evaluated the impact of COVID-19 infection on stroke care among 457 stroke centers of 70 countries [22]. The authors found that stroke admission was decreasing by 11.5% after the pandemic months. In this study, stroke admission was declined by approximately 30% as shown in Table 1. The admission rates for thrombotic, embolic and hemorrhagic stroke were lowered by 34.40%, 32.52% and 37.43% for the year 2020 compared with the year 2019. The differences between this study and the previous study may indicate that decline rate of stroke during the pandemic may vary among countries [22]. Other than decreasing of stroke admission during the pandemic period, we also found that only thrombotic and hemorrhagic stroke subtypes were significantly decreasing in the post-outbreak period. This study has the main strength of large study population with various types of stroke. Additionally, we collected data from the reimbursement database. Therefore, an issue of completeness of data may be fewer than the previous study [22].

There are some limitations in this study. First, the intervention in this study was COVID-19 outbreak as a quasi-experimental study. This type of study may have little value than randomized controlled trial but it cannot be performed in this pandemic. Second, personal data such as co-morbid diseases were not studied as well as other interventions [23–27]. Stroke type is defined based on the diagnosis by an attending physician. Finally, no personal data of each patient were studied as well as the laboratory results as this study evaluate the effects of COVID-19 outbreak on national incidence rates of each stroke subtype. These data were beyond the scope of the study. Our data may not represent all stroke population in the country as they were stroke patients admitted to other databases or out of hospitals. However, this database covers for 75% of the country population.

In conclusion, the incidence rates of three stroke subtypes were increasing prior to the COVID-19 outbreak. The COVID-19 outbreak significantly impacts hospital admission rates of both thrombotic and hemorrhagic stroke subtypes.
